# Model-Assisted Estimation of the Genetic Variability in Physiological Parameters Related to Tomato Fruit Growth under Contrasted Water Conditions

**DOI:** 10.3389/fpls.2016.01841

**Published:** 2016-12-09

**Authors:** Dario Constantinescu, Mohamed-Mahmoud Memmah, Gilles Vercambre, Michel Génard, Valentina Baldazzi, Mathilde Causse, Elise Albert, Béatrice Brunel, Pierre Valsesia, Nadia Bertin

**Affiliations:** ^1^Plantes et Systèmes de Culture Horticoles, Institut National de la Recherche Agronomique - Centre PACAAvignon, France; ^2^Unité Génétique et Amélioration des Fruits et Légumes, Institut National de la Recherche Agronomique – Centre PACAMontfavet, France

**Keywords:** fleshy fruit, quality, ideotype, *Solanum lycopersicum*, virtual fruit model, water stress, multiobjective optimization

## Abstract

Drought stress is a major abiotic stress threatening plant and crop productivity. In case of fleshy fruits, understanding mechanisms governing water and carbon accumulations and identifying genes, QTLs and phenotypes, that will enable trade-offs between fruit growth and quality under Water Deficit (WD) condition is a crucial challenge for breeders and growers. In the present work, 117 recombinant inbred lines of a population of *Solanum lycopersicum* were phenotyped under control and WD conditions. Plant water status, fruit growth and composition were measured and data were used to calibrate a process-based model describing water and carbon fluxes in a growing fruit as a function of plant and environment. Eight genotype-dependent model parameters were estimated using a multiobjective evolutionary algorithm in order to minimize the prediction errors of fruit dry and fresh mass throughout fruit development. WD increased the fruit dry matter content (up to 85%) and decreased its fresh weight (up to 60%), big fruit size genotypes being the most sensitive. The mean normalized root mean squared errors of the predictions ranged between 16–18% in the population. Variability in model genotypic parameters allowed us to explore diverse genetic strategies in response to WD. An interesting group of genotypes could be discriminated in which (i) the low loss of fresh mass under WD was associated with high active uptake of sugars and low value of the maximum cell wall extensibility, and (ii) the high dry matter content in control treatment (C) was associated with a slow decrease of mass flow. Using 501 SNP markers genotyped across the genome, a QTL analysis of model parameters allowed to detect three main QTLs related to xylem and phloem conductivities, on chromosomes 2, 4, and 8. The model was then applied to design ideotypes with high dry matter content in C condition and low fresh mass loss in WD condition. The ideotypes outperformed the RILs especially for large and medium fruit-size genotypes, by combining high pedicel conductance and high active uptake of sugars. Interestingly, five small fruit-size RILs were close to the selected ideotypes, and likely bear interesting traits and alleles for adaptation to WD.

## Introduction

Drought stress is one of the major abiotic stresses, which represents the primary cause of crop loss worldwide, and the development of more water efficient cropping systems is becoming critical (Bodner et al., 2015). Nonetheless, in the case of fleshy fruits, moderate drought has been suggested to improve both organoleptic quality and nutritive value (Ripoll et al., 2014). Trade-offs between quality and yield seem realistic, but depend strongly on stress intensity and genotypes (Ripoll et al., 2016). Indeed, recent studies on tomato revealed a strong genetic variability in the response to drought from negative to nil to positive impact on fruit size and quality (Ripoll et al., 2015). A large number of genes and molecular mechanisms involved in survival under drought have been identified, in particular in *Arabidopsis thaliana* (L.) Heynh. (Blum, 2011). These genes are involved in the control of many physiological processes, but they do not necessarily confer a stress resistance and they may entail detrimental effects on yield and quality in crop plants facing long periods of drought combined with high temperature (Gong et al., 2010; Tardieu, 2012). In tomato, only a few QTLs/genes involved in the response to water deficit are known (Labate et al., 2009). In a recent study (Albert et al., 2016), a RIL population of 117 F7 recombinant inbred tomato lines has been genotyped for 501 SNP markers and phenotyped under control (C) and water deficit (WD). This study revealed a total of 56 QTLs of plant and fruit traits, among which 11 depended on watering regime. Interestingly, these authors observed a large genetic diversity in plant and fruit responses to WD and significant genotype by watering regime interactions, suggesting the possibility to develop tomato genotypes adapted to grow under water limitation. The diversity present in genetic resources of tomato species is a vital source of traits and alleles for crops, many of which may have been inadvertently lost during selection. Thus, identifying main mechanisms governing fruit adaptation to water deficit and pinpointing genes, QTLs and phenotypes that will enable a fruit to maintain growth and improve quality under conditions of limited water supply is a crucial challenge for breeders and growers in the light of current issues related to climate change.

Crop models are adequate tools for analyzing genotype by environment interactions, since they integrate environmental and genetic effects on individual physiological processes and are able to predict interactions among processes during fruit development (Bertin et al., 2010). The Virtual Fruit Model (Fishman and Génard, 1998), an eco-physiological process-based model which describes both water and dry matter accumulation rates in fleshy fruits, has already proven its robustness and genericity under contrasted environmental conditions and for different fruit species: peach (Quilot et al., 2005), mango (Lechaudel et al., 2007), kiwifruit (Hall et al., 2013), and tomato (Liu et al., 2007). Notably, this model has been used to assess water deficit impacts on fruit growth (Lescourret and Génard, 2005; Baldazzi et al., 2013). In such mechanistic models, the parameters are linked to physiological traits or processes which can be linked to loci or genes. Each parameter is in fact related to a set of interconnected processes controlled by a group of genes, which was defined by Tardieu (2003) as “meta-mechanism.” Though plant traits generally depend on genotype, environment and cultural practices, model parameters should be, ideally, independent of the environment and management. Some of these parameters,—called genotypic parameters,—are genotype dependent while others are generic and independent of the genotype (Boote et al., 2001). The set of genotypic parameters related to a particular genotype represents a phenotypic fingerprint of this genotype and it is amenable to QTL analysis (Bertin et al., 2010). Several attempts have been made to include genetic information into process-based models and to link model parameters to genes or QTLs (White and Hoogenboom, 1996; Chapman et al., 2003; Reymond et al., 2003; Quilot et al., 2005; Xu et al., 2012; Rebolledo et al., 2015). The main difficulty is that the model should capture sufficient physiological functionalities, to simulate the expression of single genes or a gene network.

An ultimate goal is then to use these enriched process-based models for the design of ideotypes adapted to biotic and abiotic stress environments. Here, an ideotype designates a “plant model which is expected to perform or behave in a predictable manner within a defined environment.” However, the fitness landscape (objectives space) to be explored to design ideotypes is often very complex and a large number of parameter combinations must be evaluated in order to identify the best-adapted genotypes. This difficulty comes from the nonlinear and non-convex nature of antagonist criteria and the complex nature of the process-based models, such as the “Virtual Fruit.” Consequently, the model-based design of ideotypes is a difficult nonlinear multi-objective optimization problem that resists to the classical simulation and optimization methods. To deal with such multi-objective optimization problems, nature-inspired optimization algorithms (e.g., genetic algorithms and particle swarm optimization algorithms) are suitable and increasingly used. Multi-Objective Evolutionary Algorithms (MOEAs) are amongst the best-known and most effective nature inspired optimization algorithms. They allow exploring high dimensional solution spaces and they do not require any derivative information. MOEAs generate many feasible and non-dominated solutions, i.e., elements of the Pareto optimal set (best tradeoffs between conflicting objectives). Many papers have been published on the use of evolutionary algorithms for ideotype model-based design. For the sake of conciseness, we mention only the works of Letort et al. (2008) on beech trees, of Qi et al. (2010) on maize, of Lu et al. (2012) on wheat, of Quilot-Turion et al. (2012) and Sidi et al. (2014) on peach, and of Ding et al. (2016) on rice.

In the present study our objectives were to use the Virtual Fruit Model (i) to phenotype a RIL population of tomatoes at the process level; (ii) to better understand the fruit growth mechanisms (water and dry matter accumulation) involved in the response to water deficit; (iii) to look for optimized sets of genotypic parameters/genotypes which could reduce the loss of fruit fresh weight under WD and at the same time maintain/improve high fruit dry matter content. The RIL population is the one previously genotyped by Albert et al. (2016). The genetic variability in fruit traits and model parameters was analyzed by Principal Component Analysis (PCA) and through QTL analysis. This step helped to explore diverse genetic strategies in response to water deficit and to discuss potential processes/genes involved in this response. Then the model was applied to design ideotypes in terms of fruit size and quality.

## Materials and methods

### RIL population and experimental design

**Figure 1:**
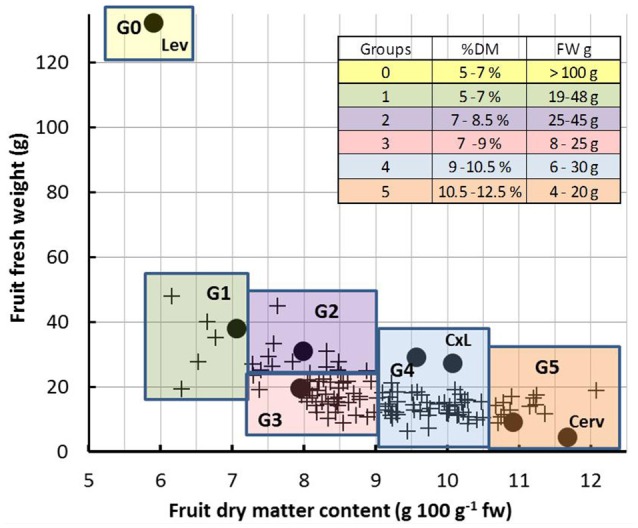
**Relationship between fruit fresh weight and dry matter content of ripe fruits under control condition**. Each symbol represents one genotype (means of 15 to 20 fruits). Black dots indicate the five representative genotypes, the two parents (Lev and Cerv) and the F1 hybrid (CxL). The colored squares represent the six groups of genotypes (G1 to G6) which were considered for model inputs. The insert gives the ranges of fresh weight (FW) and dry matter content (%dm) of ripe fruits in each group.

The RIL population, including 117 F7 recombinant inbred lines, was developed from an intraspecific cross between two inbred lines, Cervil and Levovil (described in Saliba-Colombani et al., 2001). Cervil is a cherry type tomato (*S. lycopersicum* var. *cerasiforme*, 6–10 g), whereas Levovil (*S. lycopersicum*) is a large fruited accession (90–160 g). The 117 RILs, the F1 hybrid and the two parents were grown in a heated glasshouse in INRA Avignon (France) from March to July 2013. Based on previous data, eight genotypes were selected in the population in order to have a good representation of the ranges in fruit size and dry matter content. These eight genotypes included the two parents and the F1 hybrid (CxL). Some input parameters of the Virtual Fruit Model (initial fresh and dry weights, fruit surface conductance to water, stem water potential) were accurately measured on these eight representative genotypes and then the same values were applied to all genotypes of the group (see below and Figure 1).

Plants were grown in 4 l plastic pots filled with peat (Klasmann 165) and watered with nutritive solution (2, 4, 6 mmol l−1, N, P, and K, respectively). All trusses were pollinated with an electrical bee. The number of flowers per truss was regulated to get homogeneous fruit load and comparable source:sink ratios among plants of a given genotype. The first two trusses of the small fruit genotypes (final fruit size <30g) were pruned to 8 fruits and the following trusses to 12 fruits. Regarding the medium and large fruit genotypes (final fruit size >30g), the first two trusses were pruned to 4 fruits and the following trusses to 6 fruits. Climate conditions (temperature, humidity and light intensity) in the glasshouse were recorded every minute and data were averaged hourly throughout the experiment.

From anthesis of the second truss of Cervil (considered as a reference early genotype), two irrigation treatments were applied: control (C) and water deficit (WD). Control plants were irrigated in order to get drainage around 25%. In the WD treatment, water supply was reduced by 64% compared to the control, corresponding to 49% of the potential evapotranspiration on average over the experimental period. The peat substrate humidity was assessed continuously with 12 small soil moisture sensors (EC-5 Decagon devices, USA) inserted in the substrate and randomly distributed in the glasshouse, and twice a week with a water content sensor (WCM-control, Grodan, Roermond, The Netherlands). Peat substrate humidity averaged 60–65% in control plants and 25–30% in WD plants (no drainage). Within the glasshouse, irrigation treatments were applied by row, and the genotypes were randomized within rows. Two plants of each genotype (10 for the parent lines and for the six representative genotypes) were grown under each treatment. The trial plants were surrounded with one row of border tomato plants.

### Phenotypic measurements

Stem water potential was measured using a pressure chamber (SAM Précis 2000 Gradignan, France) at predawn and at solar noon. Measurements were performed twice during the stress period on five plants of the eight representative genotypes under both conditions. The fruit conductance was measured on three ripe fruits of the eight genotypes in both treatments, according to the weight loss method described in Lescourret et al. (2001). Flower anthesis was recorded on four successive trusses on all plants (excluding the first two trusses). The fruit fresh and dry masses were measured from 8 days after anthesis (daa) until fruit ripening (from beginning of June to beginning of July) on the whole population. About 4–5 fruits were sampled every 7 days for the 8 representative genotypes. For all other genotypes, three fruits were sampled at 8–10 daa, 12–15 daa, and 20–25 daa. At ripening about 15 to 20 fruits were sampled on all genotypes. For a given developmental stage, fruits were sampled on trusses which developed during the same time window. Within a truss, the first proximal and last distal fruits were not sampled, in order to avoid fruit position effects. The fruit fresh mass was measured after harvest and the fruit dry mass was measured after drying in a ventilated oven for 72 h. All sampled fruits from the WD treatment were grown after water deficit onset, which means that cell division, cell expansion and ripening processes were all affected by WD.

### Virtual fruit model description

Fishman and Génard (1998) developed a biophysical model which simulates water and dry matter accumulation rates in the fruit, using as inputs two climatic variables (fruit temperature and air humidity) and two variables describing the plant status (stem water potential, and phloem sap concentration in sugars). This model describes the biophysical processes involved in fruit growth, with appropriate equations computing uptakes from the xylem and phloem across composite membranes, and losses of dry matter and water due to respiration and transpiration, respectively. Hall et al. (2013) extended the model formulation by adding a pedicel, which contributes to the major hydraulic resistance of the pathway to the fruit (Mazzeo, 2008). The extended version of Hall et al. (2013) was used in our study. Water and sugar flow from the stem through the pedicel into an intermediate compartment, that we called the fruit vasculature, and then through composite membranes into the fruit. The equations describing flows from the fruit vasculature into the fruit (*U*_*p*_ = mass flow from phloem, *U*_*x*_ = mass flow from xylem, *U*_*s*_ = sugar flow) and those describing fruit respiration (*R*_*f*_) and transpiration (*T*_*f*_) are the same as those given by Fishman and Génard (1998). The model simulates two state variables (*w* = mass of water in fruit, *s* = dry mass of fruit), whose rates of change are:

(1) $$\frac{dw}{dt}=U_{x}+U_{p}+r_{w}R_{f}-T_{f}$$

(2) $$\frac{ds}{dt}=U_{s}-R_{f}$$

where *r*_*w*_ is the proportion of dry mass converted to water during respiration (*r*_*w*_ = 9/16 according to Hall et al., 2013).

Three parallel mechanisms involved in sugar uptake (*U*_*s*_) from the phloem were considered: active uptake (using Michaelis-Menten kinetics), mass flow, and diffusion (equations are described in Liu et al., 2007).

The rate of fruit volume (*V*) increase is given by:

(3) $$\frac{dV}{dt}=\{\begin{array}{cc} V\phi \text{​}\left(P_{f}-Y\right) & P_{f}>Y\\ 0 & \text{otherwise} \end{array}$$

where ϕ and *Y* are respectively, the cell wall extensibility and yield threshold parameters of the Lockhart equation. When this is equated to the rate of volume increase calculated from the mass balance, we get an algebraic equation for *P*_*f*_ (fruit turgor pressure).

The water and carbon fluxes through the pedicel xylem (which primarily carries water) and phloem (which carries water and sugar) were considered, as in the model developed by Hall et al. (2013).

To identify the main genotypic parameters that affect the model outputs, we performed a sensitivity analysis of the “Virtual Fruit” model, which includes 30 parameters. Three sensitivity analysis methods were used for this purpose: one elementary effects method, i.e., Morris method, and two methods based on the variance decomposition, i.e., the Fourier Amplitude Sensitivity Test (FAST) and the Sobol's methods (Saltelli et al., 2008). Based on the conclusions of those methods, a cross selection of the most important parameters was performed. Accordingly, six genotypic parameters involved in different processes had significant impacts on model outputs (Table 1). Two additional parameters were chosen because of their impact on carbon and water transports, which are main processes on which this study focusses. The first one (tauS) drives the mass flow, whereas the second one (lp1) is related to the pedicel conductivity which is strongly involved in water uptake from the phloem. These eight genotypic parameters are described in Table 1.

**Table 1:** **Description of the eight genotypic parameters used in the calibration step and of the three additional parameters used for designing ideotypes**.

**Parameter name**	**Description**	**Boundaries**			
		**Calibration**		**Ideotypes design**	
		**Lower**	**Upper**	**Lower**	**Upper**
phiMax [bar^−1^ h^−1^]	Maximum cell wall extensibility. Involved in cell expansion rate	1.0E-04	0.01	0.002	0.02
Lp [g cm^−2^ bar^−1^ h^−1^]	Conductivity of the composite membrane for water transport from phloem to fruit cells	5.0E-04	0.4	0.02	0.6
nuM [gs h^−1^]	Maximum sugar active uptake rate. Involved in the sugar active uptake calculus (Ua)	0.002	0.15	0.002	0.2
tstar [h]	Involved in the sugar active uptake calculus. The higher is tstar, later the active uptake begins to decrease	10	900	10	900
tauA [h]	Involved in the sugar active uptake calculus. The higher is tauA, the slower is the active uptake decreasing rate in the growth stage	5	900	72	900
tauS [h^−2^]	Involved in the calculus of the reflection coefficient of the composite membrane (sigmaP) which increases with tauS. sigmaP is involved in phloem mass flow	5.0E-06	1.5E-05	1.5E-06	2E-05
lp1 [g bar^−1^ h^−1^]	Pedicel conductivity for the water transport in phloem	5.0E-05	0.1	0.002	0.2
rxp [dimensionless]	Rxp = Lx/Lp = Lx1/Lp1. Lx and Lx1 have the same meaning as Lp and Lp1 but they refer to the xylem	0.1	0.6	0.1	0.8
s0 [g]^1^	Initial fruit dry weight			0.019	0.086
w0 [g]^1^	Initial fruit water weight			0.126	1.0
bssrat [dimensionless]^1^	Involved in the soluble sugar concentration calculus. Ssrat = assrat^*^t/24 + bssrat Ssrat is the ratio between soluble sugars mass and the total dry mass			0.043	0.22

*1 = irrigation dependent parameter, optimized only for the ideotype design*.

*The parameter ranges used in the calibration step are based on literature data. The lower boundaries of phiMax, lp, and lp1 are set near to 0 for computational stability reasons; tauA, tstar, and tauS are based on experimental information on fruit development*.

### Model calibration

As mentioned above, the model genotypic parameters were assumed to be genotype dependent and environment independent, i.e., they do not depend on the irrigation conditions. Thus, each set of parameters is a footprint of one of the 117 tomato RILs. To account for the different plant and fruit status under C and WD conditions, some of the model inputs were measured experimentally under each treatment: the stem water potential, the fruit surface conductivity to water vapor, the initial dry and fresh masses, and the fruit osmotic pressure related to soluble compounds other than sugars.

The model calibration aims at estimating the values of the eight selected genotypic parameters in order to minimize the fitting errors (observed vs. simulated fruit fresh and dry weights) for each genotype. The performance index used in the model calibration was the Normalized Root Mean Squared Error (NRMSE), a dimensionless indicator that takes into account the time steps in which more observations were available along with fewer observations at other time steps. This index is suggested in Wallach et al. (2013):

(4) $$NRMSE\;[\%]=100^{*}\frac{\sqrt{\frac{1}{\text{n}}*\sum_{i\;=\;1}^{\text{n}}{\left(O_{i}-S_{i}\right)}^{2}}}{\frac{1}{\text{n}}*\sum_{i\;=\;1}^{\text{n}}O_{i}}$$

where Oi and Si are respectively, the observed and simulated values of fruit fresh and dry masses, and n is the number of observations.

The four objectives corresponding to the four NRMSE values, related to the fruit dry and fresh masses under C and WD conditions, were aggregated into two objectives. For this purpose the mean NRMSE value calculated under each irrigation condition was considered in order to have a balanced fitting error between the fruit weight components:

(5) $$f_{1}\left(X\right)={NRMSE_{aggr}}_{C}=\frac{{NRMSE_{f}}_{C}+{NRMSE}_{dC}}{2}$$

(6) $$f_{2}\left(X\right)={NRMSE_{aggr}}_{WD}=\frac{{NRMSE_{f}}_{WD}+{NRMSE}_{dWD}}{2}$$

where $\text{X}={\left(x_{1},\;x_{2},x_{3},\ldots ,x_{8}\;\right)}^{T}$ is the vector of parameters generating the (*f*_1_, *f*_2_) objective values. NRMSE_fC_ and NRMSE_dC_ are related to respectively, the fruit fresh weight and fruit dry weight predictions in the control (C) condition. NRMSE_fWD_ and NRMSE_dWD_ are related to respectively, the fruit fresh weight and fruit dry weight predictions in the water deficit (WD) condition.

The model calibration was therefore formulated as a multi-objective problem as follows:

(7) $$\min_{X\in D}\text{​}\left\{f_{1}\left(X\right),f_{2}\left(X\right)\right\}$$

where *D* is the search space defined by boundaries of the considered parameters. The problem solutions X^*^ are all the parameter sets belonging to the Pareto front, i.e., the set of solutions that consists in the best tradeoffs between the two conflicting objectives.

### Design of ideotypes

In this step, we aimed to design ideotypes of tomato adapted to WD conditions. The term ideotype designates a combination of genotypic parameters that represent virtual tomato genotypes with optimized tolerance to water deficit. For this purpose, we considered a set of 11 genotypic parameters, adding three new genotype dependent parameters to the search space (Table 1). In this study, the ideotype design aimed at (i) maximizing the ratio between dry weight and fresh weight at the ripe stage (dry matter content *dm*) until a maximal value of 10% under C condition, and (ii) minimizing the fresh weight loss associated with water deficit. Thus the ideotype design was formulated as a multi-objective problem as follows:

(8) $$f_{1}\left(X_{id}\right)=\;dm_{C}\;[\%]=100^{*}\frac{{dry_{weight}}_{C}}{{fresh_{weight}}_{C}}$$

(9) $$f_{2}\left(X_{id}\right)=loss\;\left[\%\right]=100^{*}\sqrt{{\left(\frac{\left({fresh_{weight}}_{C}-{fresh_{weight}}_{WD}\right)}{{fresh_{weight}}_{C}}\right)}^{2}}$$

The problem formulation becomes:

(10) $$\min_{X_{id}\in {\text{D}}_{\text{id}}}\text{​​}\left\{-f_{1}\left(X_{id}\right),f_{2}\left(X_{id}\right)\right\},\;\text{ Subject}\;\text{ to}\;{\text{ dm}}_{\text{C},\text{WD}}\left[\%\right]<10\%$$

where ***X***_*id*_ is the parameter vector belonging to the set *D*_**id**_, which represents the ideotypes search space. The negative sign of *f*_1_(***X***) objective is introduced to transform the minimization into maximization.

Because the sensitivity to WD depends on fruit size, we considered three groups of tomatoes differing by their final fresh weight in control conditions: large size (100–300 g), medium size (20–80 g) and small size (5–15 g).

### Optimization algorithm for model calibration and design of ideotypes

The NSGA-II developed by Deb et al. (2002) has proven to be one of the most efficient algorithms for solving multi-objective problems. Therefore, we used this algorithm both for the “Virtual Fruit” model calibration and for the tomato ideotype design. For sake of simplicity, we do not give a full description of this algorithm. The interested readers can refer to the above cited. The NSGA-II algorithm was applied through the Java package *jMetal*. As the NSGA-II algorithm depends on random variables, the optimization process was repeated 10 times in the calibration phase and 20 times for the ideotype design. At the end of the process, we could have high number of similar solutions. Therefore, the choice of the best compromise solution for the calibration step was based on the *min-max* decision criterion, to avoid high mean fitting errors in each condition. Therefore, among the solutions ${\text{X}}_{i}^{*}$ belonging to the Pareto-optimal solution set *P*, we chose the solution $\bar{\text{X}\;}$ that satisfied the following condition:

(11) $$\text{min}\{{\text{max}}_{X*\in P}\{f_{1}\left(X^{*}\right),f_{2}\left(X^{*}\right)\}\}$$

where *P* is the set of Pareto-optimal solutions. We also checked that among the best sets of parameters estimated for one genotype (solutions that all have similar objective values), parameters were not correlated (data not shown).

For the design of ideotypes, we performed a Principal Component Analysis on the parameter sets, whose corresponding objective values matched the following decision criteria:

(12) $$dm_{C}\left[\%\right]\geq \;8\%\;and\;loss\;\left[\%\right]\leq 15\%$$

### Principal component analysis and hierarchical clustering on PCA individuals score

A Principal Component Analysis (*ade4* package developed for the *R* software) (Dray and Dufour, 2007) was performed on the parameter values estimated for each recombinant line. This analysis was also applied to study the ideotype features. Genotypic parameters obtained for both calibration and ideotype design, were set as active variables. The dry and fresh weights under C and WD conditions and the dry matter content and fresh mass reduction under WD conditions were added as supplementary variables for the first PCA (model calibration step), while for the second (ideotype design) the initial dry weight, the initial fresh weight, and the *bssrat* parameter (contributing to the soluble sugar concentration calculus) were added. Data were previously normalized and centered (subtracting the mean value and dividing by the standard deviation). Among the 120 calibrated individuals, we excluded one outlier individual.

The PCA individual scores of the model calibration were subjected to hierarchical cluster analysis, using the *complete-linkage* clustering method with the *hclust* R function. The cluster number was chosen according to a visual criterion based on the cluster dendogram. For the ideotype analysis, the three groups of fruit size (large, medium and small size) were used to group the individual scores.

### QTL analysis of model genotypic parameters

The best estimations of the eight genotypic parameters for the 120 genotypes and the coordinates of the RILs on the three first axis of the PCA were used as phenotypic traits in the QTL detection. When distributions were skewed, the best corrections for normality were applied: LOG10(nuM); $\sqrt{tstar}$; LOG10(lp1); LOG10(lx); LOG10(lx1); 1/rxp. The QTL detection was performed as presented in Albert et al. (2016) using the genetic map developed by Pascual et al. (2016) which included 501 SNP markers covering 80% of the tomato genome. Briefly, the simple interval parametric mapping model (Lander and Botstein, 1989) based on the EM algorithm method implemented in the R/QTL package (Broman et al., 2003) was used. A 1000-permutation test was performed to estimate the significant thresholds. Firstly, a LOD threshold equal to 3.13 and corresponding to a genome wide significance level of α = 0.05 was considered. Then, we also considered lower significance levels to detect more QTLs: α = 0.10 (LOD threshold = 2.76), α = 0.20 (LOD threshold = 2.42) and α = 0.30 (LOD threshold = 2.20). For each detected QTL, position, LOD score, marker at the LOD score peak, confidence interval (CI, LOD decrease of one unit), average phenotypic values of the two parental alleles and percentage of phenotypic variation explained (PVE) were displayed. The CIs were expressed both in cM Haldane (genetic distance) and in Mbp onto the tomato genome (assembly v2.5) (physical distance). The number of genes within each interval was identified from the tomato genome annotation (2.4). We reported the locations between the detected QTLs and the QTLs identified on phenotypic traits (plant and fruit traits) measured on the same plants (see Albert et al., 2016).

## Results

### Water deficit effects on the observed dry matter content and fresh weight

**Figure 2:**
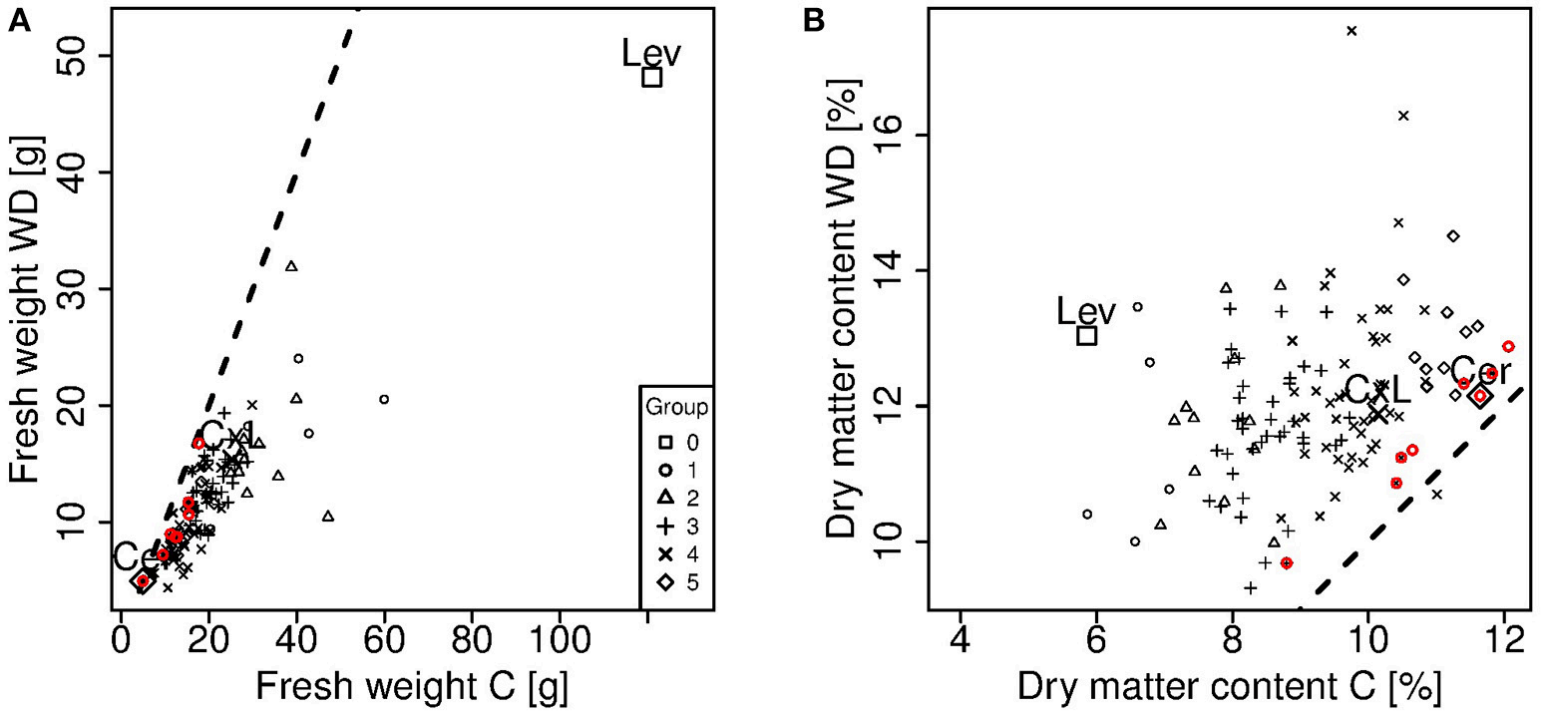
**Comparison of fresh weight (A)** and dry matter content **(B)** measured on ripe fruits under control (C) and water deficit (WD) conditions. Each point represents one genotype (means of 15 to 20 fruits) and crosses indicate the parental lines (Cervil and Levovil) and the F1 hybrid (CxL). The different symbols represent the six groups of genotypes shown in Figure 1. The dashed lines represent the condition in which the plotted variables are equal. The red points represent the genotypes that are near to both dashed lines. These genotypes are the same in **A** and **B**.

The observed values of fresh weight and dry matter content measured at the ripe stage under control and WD conditions are shown on Figures 1, 2. WD generally increased the dry matter content (up to 85%) and decreased the fruit fresh weight (up to 60%). This was directly connected to the lower influx of water to the fruit under WD conditions. Cervil—characterized by a low fresh weight—was the less sensitive to WD. The dry matter content of the F1 hybrid (CxL) increased substantially, while its fresh weight decreased slightly. On the contrary, Levovil was the most sensitive to WD, since it lost more than half of its fresh weight and it doubled its dry matter content under WD. In the population, the relative decrease in fruit fresh weight under WD was negatively correlated to the fresh weight under control conditions, indicating that large fruit genotypes were the most sensitive to WD, as mentioned in Albert et al. (2016). On the contrary, the increase in dry matter content under WD was rather independent of the dry matter content observed under control condition. Interestingly a few genotypes were close to the bisector and thus, get comparable fresh weight or dry matter content under both conditions. For these genotypes (Cervil, SSD12, SSD17, SSD49, SSD61, SSD65, SSD140, and SSD154), the differences between C and WD conditions was less than 5 g fresh mass and 1% dry matter content (Figure 2).

### Model calibration and genetic variability in model genotypic parameters in the RIL population

Eight genotypic parameters of the model (Table 1) were estimated for the RILs and for the two parent lines, in order to predict the dry and fresh masses (output variables) during fruit growth. The fittings were fairly good. Table 2 shows the NRMSE values obtained under C and WD conditions that were obtained for the whole population, for the parental lines and for the F1 hybrid. Considering the dry and fresh mass increases over the developmental period (from 8 daa to maturity), the mean NRMSE of the population ranged between 16 and 18% (standard deviation ~ 4–5%) whatever the condition and output variables (Table 2). The total variation of NRMSE values in the population was in the range of 5–34%. NRMSE values obtained for Cervil were close to the minimum for all objectives. The dry mass increase of Levovil fruits was better simulated in C than in WD condition, while the prediction of their fresh mass increase was the worst under C condition.

**Table 2:** **Statistical summary of the Normalized Relative Mean Squared Errors (NRMSE) obtained with the model calibration under control (C) and water deficit (WD) conditions**.

**NRMSE**	**Mean [%]**	**Standard deviation [%]**	**Minimum [%]**	**Maximum [%]**	**Parents and F1 [%]**	
Fresh weight in C condition	17.41	5.35	7.88	34.00	Cer	8.65
					CxL	16.36
					Lev	34.00
Dry weight in C condition	16.48	4.03	8.61	28.18	Cer	9.08
					CxL	8.72
					Lev	15.78
Fresh weight in WD condition	17.76	4.10	9.34	34.20	Cer	9.42
					CxL	12.88
					Lev	24.11
Dry weight in WD condition	17.65	4.19	8.29	27.46	Cer	8.29
					CxL	12.70
					Lev	25.19

*The dry and fresh weight increases were fitted from 8 daa until fruit maturation and NRMSE were calculated over this developmental period for each genotype and condition. Mean and standard deviations were calculated for the whole RIL population (including the parent lines). Minimum and maximum refer to the lower and upper values of NRMSE obtained in the population. On the last column, the parents and the F1 hybrid values are shown*.

**Figure 3:**
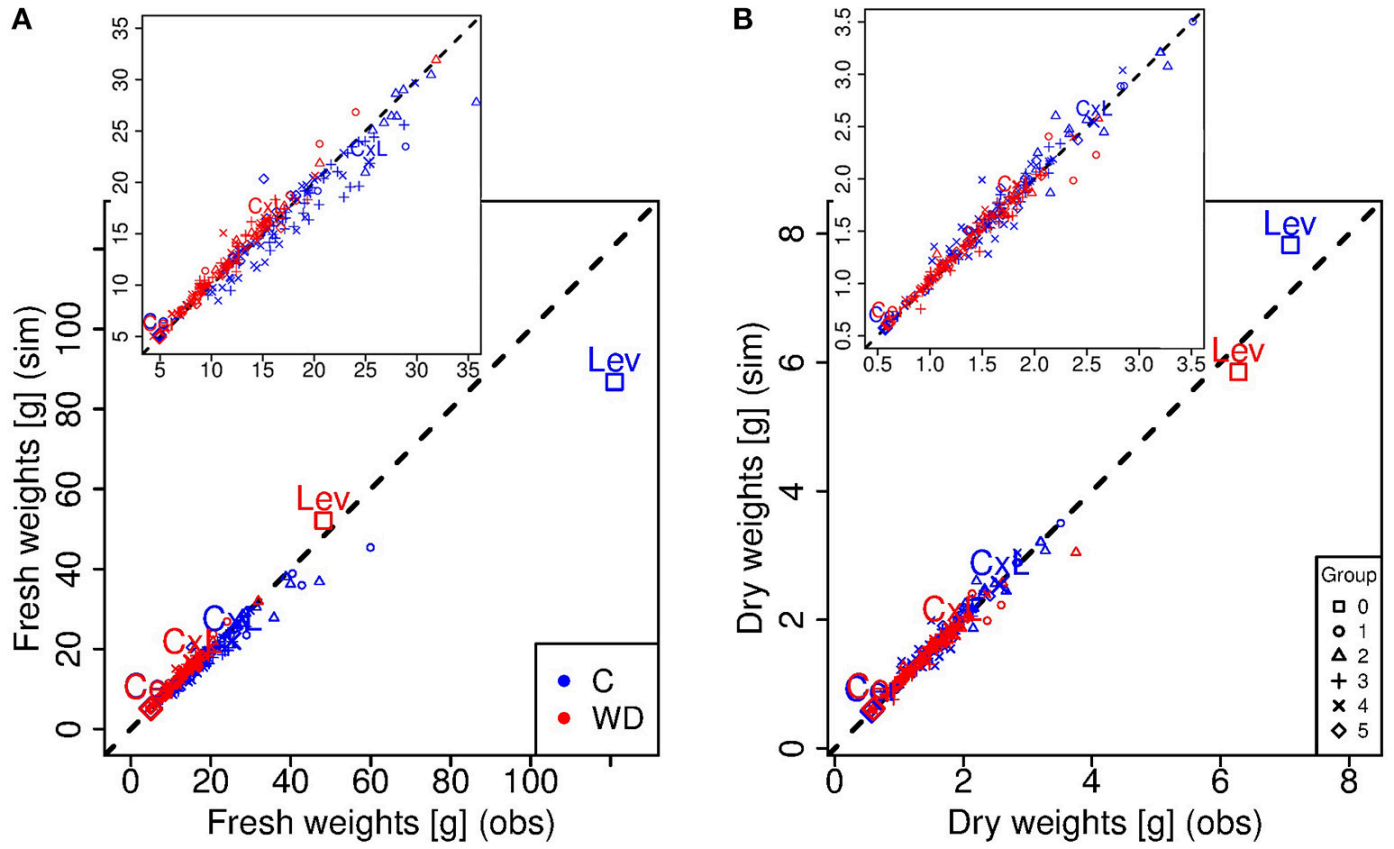
**Simulated (y-axis) *versus* observed (x-axis) fresh (A)** and dry **(B)** weight values at the red ripe stage for each genotype in control (C, blue) and water deficit (WD, red) conditions. Each point represents one genotype (means of 15 to 20 fruits) and crosses indicate the parental lines (Cervil and Levovil) and the F1 hybrid (CxL). The different symbols represent the six groups of genotypes shown in Figure 1. The dashed line represents the points in which the simulated values are equal to the observed one.

**Figure 4:**
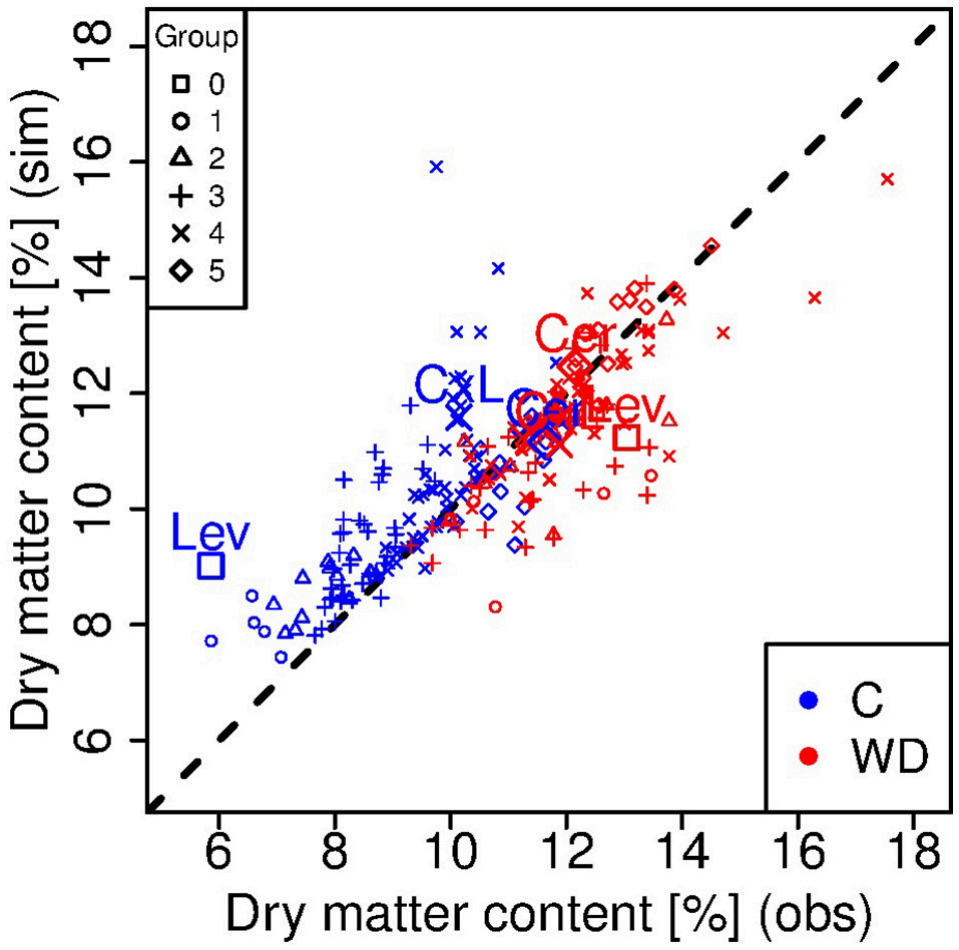
**Observed *vs*. simulated dry matter contents at the red ripe stage for each genotype in control (C, blue) and water deficit (WD, red) conditions**. Each point represents one genotype (means of 15 to 20 fruits) and crosses indicate the parental lines (Cervil and Levovil) and the F1 hybrid (CxL). The different symbols represent the six groups of genotypes shown in Figure 1. The dashed line represents the points in which the simulated values are equal to the observed one.

Considering the ripe stage, the final fruit dry mass was more accurately predicted by the model than the final fresh mass (Figure 3), and predictions were better under WD than C condition. Indeed, the model underestimated the fresh mass of the largest fruit-size genotypes in C condition, in particular for Levovil (−34 g). For this genotype, the prediction errors of fresh mass in C conditions were high over the whole development period, as indicated by the NRMSE value in Table 2. On the contrary, the model predictions were more accurate for Cervil and the CxL hybrid under both conditions. As a consequence, the final dry matter content was mostly overestimated (differences from −1.73 to 6.17%) and underestimated (differences from −3.16 to 1.37%) in C and WD conditions, respectively (Figure 4).

**Figure 5:**
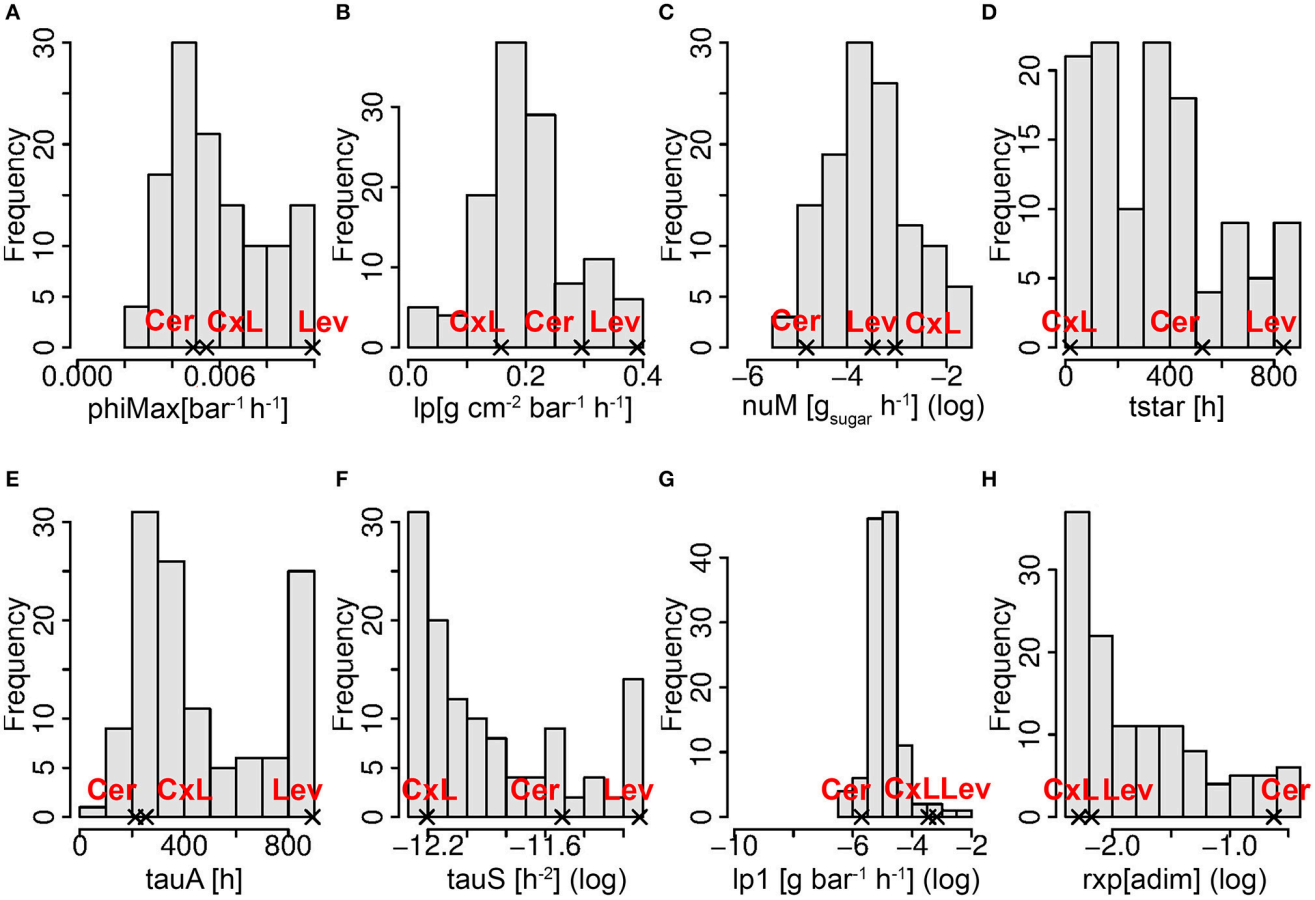
**Frequency distributions of the best estimated parameter sets in the RIL population including the two parental lines (Cer: cervil and Lev: levovil), the F1 hybrid (CxL), and the 117 RILs**. Panels **(A–H)** represent the frequency distributions of the eight genotypic parameters as indicated in the x-axes title. The x-axes range corresponds to the parameter search space (see Table 1). The log in brackets indicates that a natural logarithmic transformation was applied on the variable, for a better legibility.

**Figure 6:**
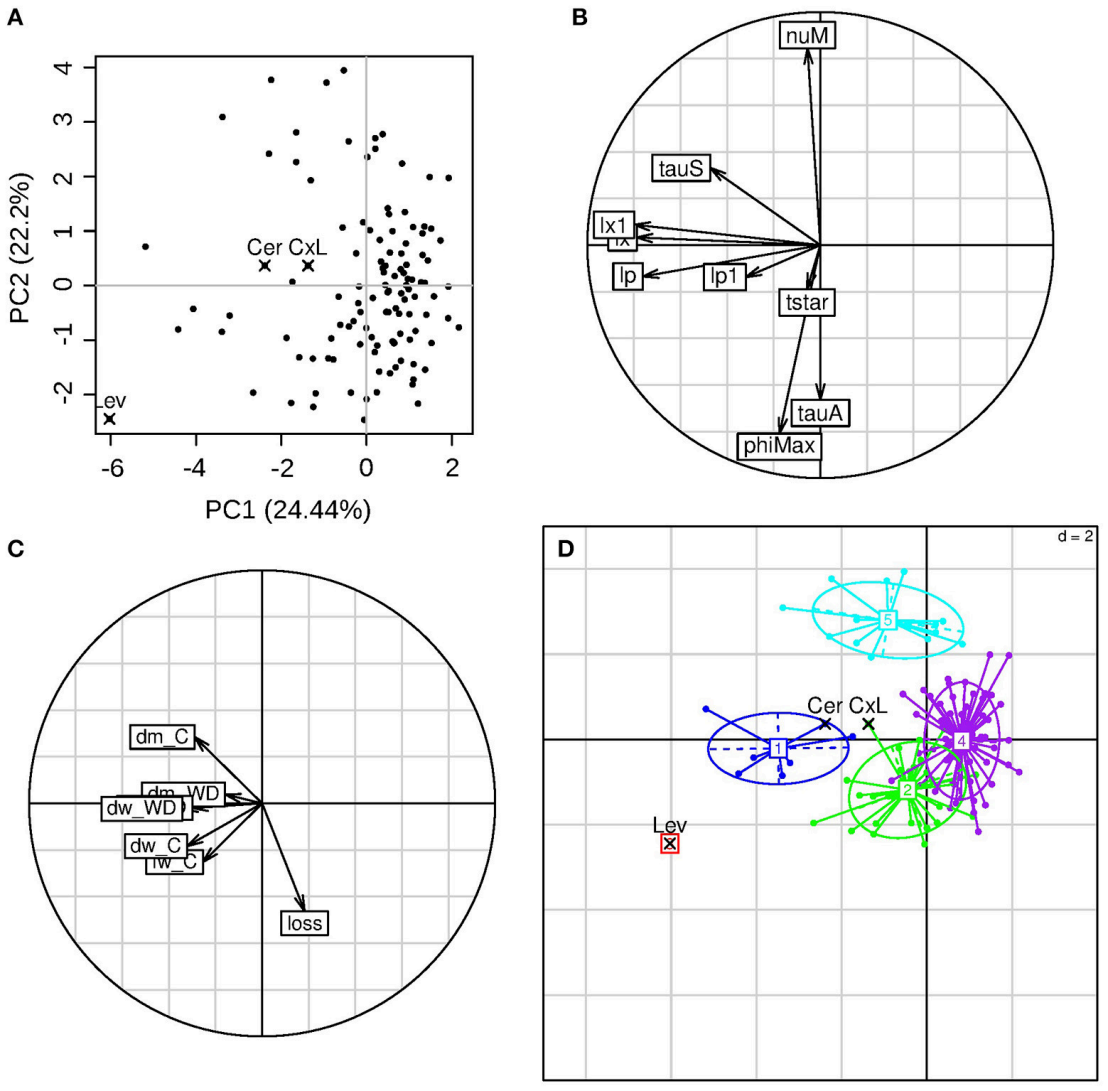
**PCA of the calibration parameter results. (A)** Individual's positions in the first two principal components plane, with highlighted values for the parental lines (Cervil and Levovil) and F1 hybrid (CxL); each component explained variance is showed in parenthesis. **(B)** Variable coordinates normed to the square root of the eigenvalue represented in the correlation circle. **(C)** Inactive variables coordinates in the correlation circle: *dm* indicates the dry matter content, *dw* and *fw* state for dry and fresh weights of ripe fruits, respectively, *loss* indicates the fresh mass loss index as computed in Equation (11); **(D)** Output clusters from the hierarchical cluster analysis.

The frequency distributions of the eight genotypic parameters were widely spread over the parameter search spaces, except for lp1 (pedicel conductivity for water transport in the phloem), which varied in a narrow range in the population (Figure 5). A principal component analysis was performed on the estimated parameter values. The first three components explained 24, 22, and 16% of the variance, respectively (62% in total). On the first principal component (Figure 6B), we observed negative loadings of lx and lp—which are the parameters related to the membrane permeability—and lx1 and lp1—related to the pedicel hydraulic conductance (Table 1). So the first axis was mainly associated with parameters controlling water inflow to the fruit from the xylem and phloem tissues. PhiMax which impacts the cell wall plasticity, as well as tauA, nuM, and tauS, which tunes the active sugar uptake and the sugar mass flow intensities, respectively, had a high loading on the second principal component. Parameter phiMax had the highest impact with a negative value, opposite with respect to nuM. So the second axis was mainly associated to turgor-driven cell expansion and active sugar uptake.

The fruit dry and fresh masses, the dry matter content under C and WD conditions, and the fresh mass reduction under WD were projected as inactive variables on the correlation circle (Figure 6C). Dry matter content in C condition and fresh mass reduction in WD condition correlated negatively. High dry matter content in C condition was associated with high value of tauS (parameter referring to mass flow process). The fresh mass reduction was associated with low values of nuM (maximum active uptake of sugar) and tauS, while it was associated with high values of phiMax (cell wall extensibility) and tauA (whose high values mean a slower decrease of active uptake rate of sugar during fruit development). Five clusters were selected through hierarchical cluster analysis (Figure 6D). The two parental lines and the F1 hybrid belonged to different groups. Levovil constituted a single cluster characterized by high fresh weight (fw) and dry weight (dw) under both conditions (first axis) and high reduction in fresh mass (second axis), with high values of lp (on the first component), phiMax and tauA (on the second component). Cluster 1 (including Cervil) and cluster 5 were characterized by high dm content under C conditions and low loss of fresh mass under WD, despite the fact they were associated with different active variables. Cluster 1 was associated with high membrane-permeability and high pedicel-conductance, whereas cluster 5 was associated with high values of nuM and low values of phiMax and tauA. Clusters 2 and 4 overlapped near to the origin of the first two components plan and were characterized by a high loss of fresh mass under WD and low dry matter content under control conditions. The CxL F1 hybrid belonged to cluster 2 and was positioned far from the cluster center.

### QTL of model genotypic parameters in the population

A QTL analysis of the eight model parameters (Table 1, best estimation for each RIL line) and the coordinates of the RIL on the PCA axes, was performed independently of the irrigation level. Results are presented in Table 3. Two QTLs were detected with a genome wide significance level of α = 0.05, on chromosome 2 and 8. These QTLs were associated with lp1 (pedicel conductivity) and lp (composite membrane conductivity) and explained 14 and 13% of the trait variations, respectively (Table 3). When considering less stringent significance levels, six supplementary QTLs became significant which explained between 9 and 11% of the parameter variations. Three of them were related to conductivity (lx1, rxp and lx, α between 0.10 and 0.30), one was related to sugar active uptake (nuM, α between 0.20 and 0.30), and two QTLs were associated with the second and third axes of the PCA (α between 0.10 and 0.20). No significant QTL was detected for phiMax, a parameter associated with cell wall extensibility, even when considering lowered significance thresholds.

**Table 3:** **QTLs detected on eight genotypic parameters of the model and on the first three axes of the PCA estimated on the RIL population**.

**Trait**	**Sign**.	**LOD**	**Chr**	**Pos**	**Marker**	**CI cM (Mpb)**	**Nb genes**	**Mean Cer (sd)**	**Mean Lev (sd)**	**PVE**	Coloc. (Albert et al., 2016)^**^
lp1^*^	0.05	3.85	2	95.60	TG167_Y02_52393366	89.73–107.19 (51.19 –54.79)	480	0.01	0.01	13.75	Nbfruits.C&WD
								(0.00)	(0.00)		fw.C&WD FIR.WD
											FIR.WD dw.C SSC.Int
rxp^*^	0.20	2.68	4	36.73	Y04_03230589	33.63–52.44	192	0.17 (0.12)	0.22	10.22	∅
						(0.30–0.48)		(0.12)	(0.13)		
lx1^*^	0.10	2.81	4	61.27	Y04_53862540	2.06–63.34	1604	0.001	0.002	10.7	FIR.C and WD
						(0.42–55.37)		(0.00)	(0.00)		
Axis3	0.20	2.46	4	86.96	Y04_61146494	61.27–95.70	589	−0.36	0.26	9.37	Flw.C
						(53.86–62.08)	589	(0.12)	(0.13)		
											Flw.WD
											Diam.C
											fw.C
											FIR.C&WD
											dw.C
											VitCFM.C&WD
											Yield.C
nuM	0.30	2.30	7	93.31	Y07_67908188	82.11–93.31	408	0.05	0.03	8.80	∅
						(65.13–67.90)		(0.01)	(0.00)		
Axis2	0.20	2.49	7	88.00	Y07_64327204	73.63–93.31	575	0.51	−0.44	9.15	∅
						(63.64–67.90)		(0.20)	(0.19)		
lp	0.05	3.31	8	42.12	Y08_57208257	31.67–58.97 (54.32–59.92)	479	0.22 (0.08)	0.16	12.64	pH.WD VitCFM.WD
									(0.06)		VitCDM.C&WD
lx^*^	0.30	2.24	8	42.12	Y08_57208257	31.67–101.95 (54.32–65.60)	1180	0.51 (0.05)	0.03	8.71	Flw.WD
									(0.01)		pH.WD VitCFM.WD
											VitCDM.C&WD
											Yield.Int

* *Traits transformed to ensure a normal distribution, LOG10(lp1); 1/rxp; LOG10(lx1); LOG10(lx)*.

** *Nbfruits, plant fruit number; fw, fruit fresh weight; FIR, fruit firmness; dw, fruit dry matter weight; SSC, solid soluble content; Flw, flowering time; Diam, stem diameter; pH, fruit pH; VitCFM, vitamin C content in fruit on a fresh weight basis; VitCDM, vitamin C content in fruit on a dry weight basis; Yield, fruit fresh weight per plant; C, control; WD, water deficit; Int, interactive between watering regimes*.

*“Sign.” indicates the significance threshold at which the QTL was detected. LOD is the log-likelihood at that marker. The chromosome is indicated under “Chr” and the position of the QTL is expressed in Haldane cM under “Pos.” The most closely associated marker is indicated. CI indicates the genetic confidence interval in Haldane cM calculated by LOD decrease of one unit, and its physical equivalent (Mpb) on genome assembly 2.5 between brackets. The number of genes in the QTL interval (genome annotation 2.4) is indicated. The average value of the two parental alleles (Cer and Lev, with the standard deviation between brackets) and the percentage of phenotypic variation explained by the QTL (PVE) are displayed. Colocalizations with phenotypic QTLs detected in Albert et al. (2016) in the same RIL population are indicated. C, QTL specific to the control condition; D, QTL specific to the water deficit condition; C&WD, QTL detected under both condition; Int, QTL with effect changing intensity or direction according to the watering conditions*.

Among the eight identified QTLs, two colocalized on top of chromosome 8 (for lp and lx), two in the centromeric region of chromosome 4 (for rxp and lx1) and two on top of chromosome 7 (for nuM and axis 2), which may correspond to three unique QTLs. Except for the QTL for rxp on chromosome 4 which included 192 genes over 0.18 Mbp, the QTL intervals were rather large (from 3.6 to 54.95 Mbp, including between 480 and 1180 genes). QTLs for fruit and plant traits detected in the same regions in the same population grown under control and WD conditions (Albert et al., 2016) are indicated in the last column of Table 3.

### Design of tomato ideotypes to minimize the reduction of fruit fresh mass under WD conditions and optimize fruit dry matter content under C conditions

The design of ideotypes consisted in finding sets of model parameters to match one or more objectives under a given condition (C or WD). Because we observed significant decrease in fruit fw under WD conditions and because fruit dm content is associated with high sugar and acid contents, our objectives were to maximize the dry matter content in C conditions and to minimize the fresh weight loss under the water deficit modality. Moreover, since the sensitivity to WD depends on the fruit fw (Figure 2), three classes of fruit grades were considered: large size (100–300 g), medium size (20–80 g) and small size (5–15 g).

**Figure 7:**
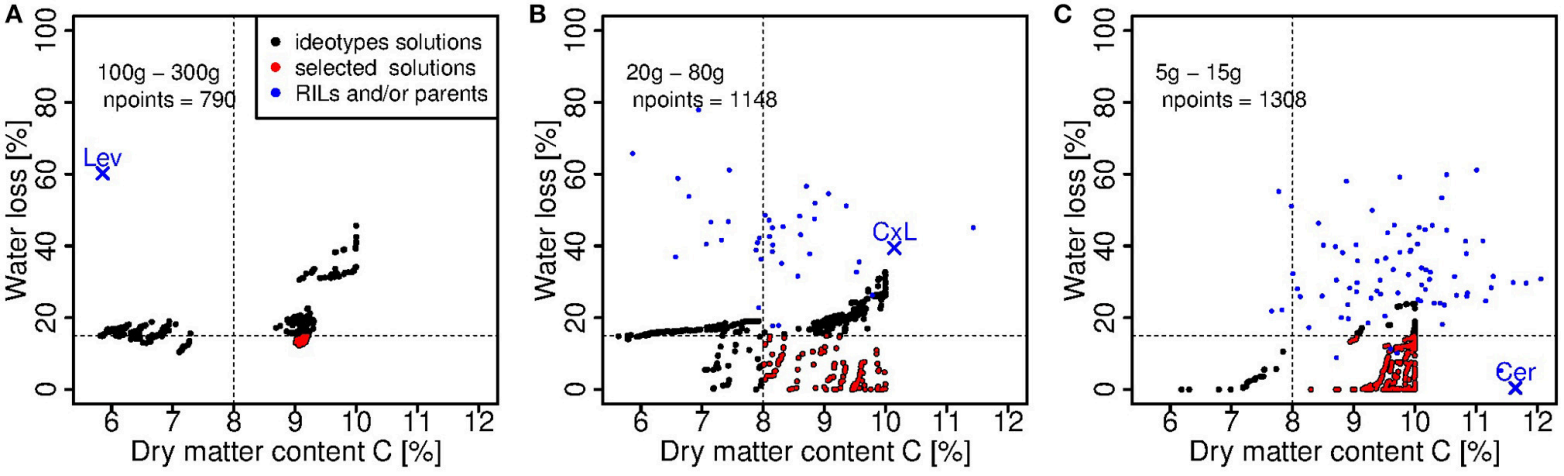
**Ideotype solutions considering a fruit fresh weight interval of (A)**: 100–300 g, **(B)**: 20–80 g, **(C)**: 5–15 g. The black and red points represent the dry matter content of ripe fruits in C conditions and the fresh mass loss value of all the ideotype solutions obtained solving the multi-objective problem for each interval of fruit fresh weight. The red points are the ideotype solutions satisfying simultaneously a high dry matter content in C condition (>8%) and a low loss of fresh mass under WD (<15%), according to Equation (13). npoints is the number of solutions. The blue points represent the dry matter content of ripe fruits in C conditions and the fresh mass loss values computed for the observed individuals in the RIL population, in the respective weight class. The parental lines (Cervil and Levovil) and F1 hybrid (CxL) are highlighted.

In this step, 11 genotypic parameters were considered (Table 1), the eight parameters estimated in the calibration step, the initial fresh and dry mass of fruit and one parameter related to sugar content. The threshold between the two objectives is highlighted on Figure 7. For the group of large fruits (Figure 7A), the objectives were largely improved (dm content around 9% under C conditions and fresh mass loss around 15% under WD) with respect to Levovil (5.5% dm content under C condition and 60% fresh mass loss under WD), the only genotype belonging to the 100–300 g interval. The median fresh weight of the ideotypes in this group was 113 g in C conditions. In the medium-size group (Figure 7B) and in the small-size group (Figure 7C) of fruits, we obtained a large improvement too, since our selected ideotypes contained between 8 and 10% dm, which was comparable to the RIL population; however, they lost less than 15% fresh mass under WD conditions, which was two to three times less than the RIL population. In these two groups, the median fresh weight in C condition was 21 and 7 g, respectively. Interestingly in the small-size group of fruits, five RILs were in the scatter plot of selected ideotypes, and likely bear interesting traits and alleles for adaptation to WD. These are Cervil, SSD84, SSD107, SSD121, and SSD154.

**Figure 8:**
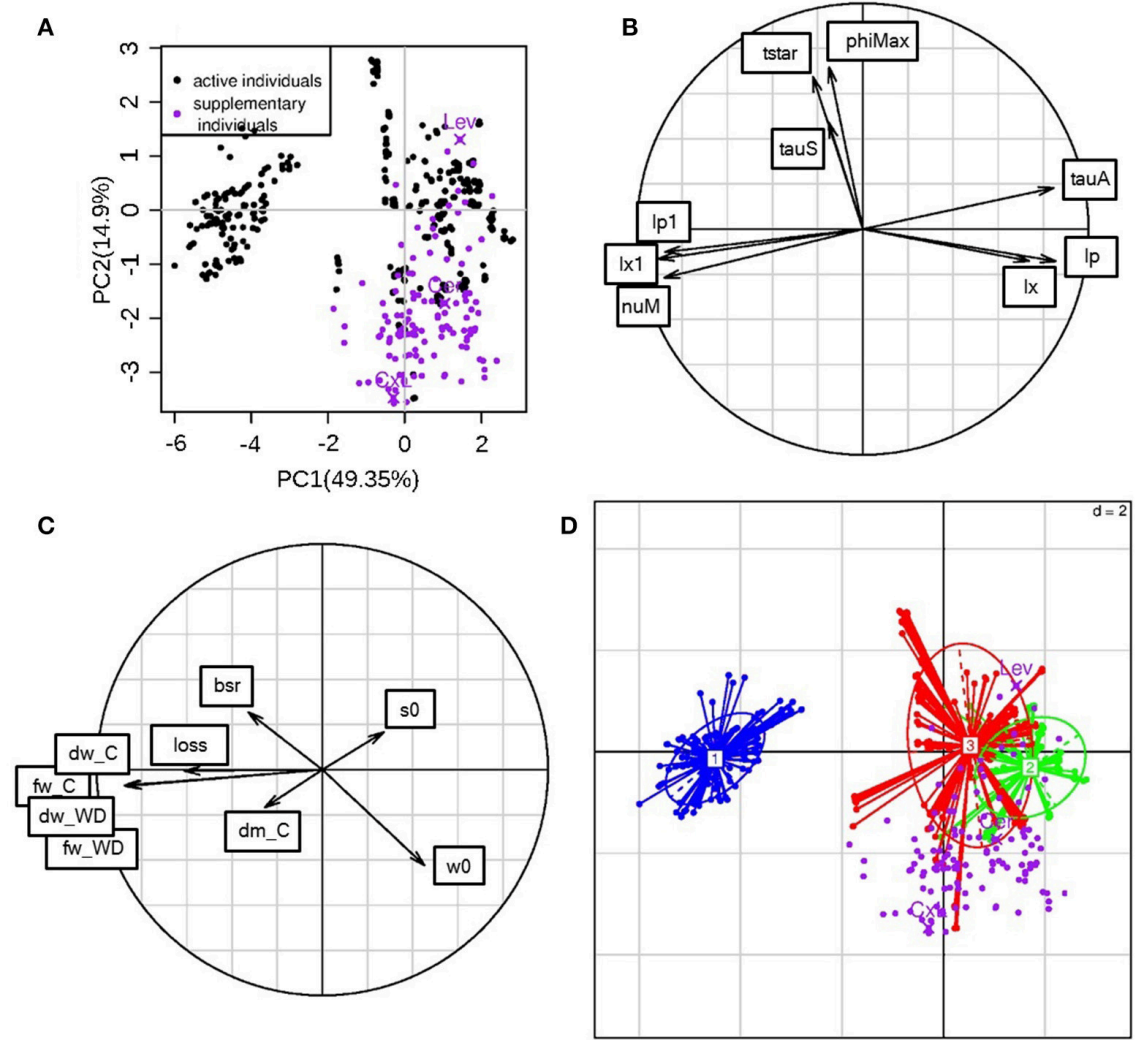
**PCA performed on the ideotype parameters**. **(A)**: ideotype positions in the first two principal components plane (black dots), and projection as inactive variables of the parameter values calibrated for the RIL population (purple dots); The parental lines (Cervil and Levovil) and F1 hybrid (CxL) are highlighted; the % of variance explained on each component is given in parenthesis. **(B)**: variable coordinates normed to the square root of the eigenvalue represented in the correlation circle. **(C)** Inactive variable coordinates in the correlation circle. *bsr* is the *bssrat* parameter, *s0* and *w0* are the estimated initial dry and fresh weights; *dm_C* states for dry matter content in C condition and *dw_C* for dry weight in C condition. Three more variables were hidden by this last one: *fw_C, fw_WD*, and *dw_WD* representing fresh weight under C and WD, and dry weight under WD, respectively **(D)**: ideotypes divided into fruit grade groups: (1) 100–300 g (blue), (2) 20–80 g (green), (3) 5–15 g (red). Purple dots represent the projections of the RILs.

A PCA was performed on the ideotype parameters obtained through the optimization problem resolution in order to understand the mechanisms of water stress resistance that could be combined in “ideal” fruits. In order to compare the ideotypes and the RILs, the eight genotypic parameters calibrated on the RILs were used as active variables (Figure 8B). The three additional parameters estimated for the ideotypes (bssrat, s0, w0) as well as the calculated fresh mass loss under WD, the fresh and dry mass and the dm content under non limiting water supply were projected as inactive variables (Figure 8C). The first two principal components explained 49.4 and 14.9% of the variation, respectively (Figure 8A). On the first component, lx and lp (composite membrane conductivity) had a positive loading and lx1 and lp1 (pedicel conductivities) had a negative loading. nuM (maximum rate of active sugar uptake) and tauA (negatively linked to the decrease in active sugar uptake rate during the fruit development) showed a negative and a positive loading, respectively, on this first component. The three groups of fruit grades were well-separated in the PC space especially for the big-size fruits (Figure 8D, cluster 1 blue colored). The big fruit-size ideotypes (100–300 g) were associated with high value of nuM, high pedicel conductivity and low fruit composite membrane conductivity, suggesting that sugar transport and pedicel conductivity may be interesting issues for improving adaptation of large-fruited genotypes to WD. They were also associated with high fresh mass loss (considered as inactive variable; Figure 8C). On the contrary, the cell wall extensibility (phiMax) and the mass flow characteristics (tauS) did not strongly discriminate the ideotype population.

The active variables were correlated in a different way with respect to the calibration situation (Figure 6A). In the ideotype principal component space, tstar and tauS correlated to each other, whereas in the calibration parameters space they were uncorrelated. Parameters regarding conductivities were highly correlated in both spaces; however, in the ideotype situation, the pedicel conductivities lp1 and lx1 were negatively correlated with the composite membrane ones (lp and lx). nuM did not show any positive correlation in the calibration case, whereas it correlated positively with the pedicel conductivities in the ideotype case. Most of the RILs, when projected as inactive individuals (Figure 8A), lied for in the positive values of the first principal component and in the negative values of the second principal component. Most of the RILs have medium to small-sized fruits (Figure 2), but, in the ideotype principal component plan, their positions did not completely overlap the corresponding group of ideotypes (n°2). Levovil is the only large fruited line and it did not belong to ideotype group 1. Cervil belonged to its size group. CxL belonged to a region that is the farthest one to group 2.

## Discussion

### Model-based analysis of the processes involved in genetic variability of fruit response to water deficit

In this study, we applied a long and severe WD, which caused significant decrease in fruit fresh mass and increase in dm content for most of the RILs. Large fruits were the most sensitive in terms of fresh mass reduction, in agreement with previous studies (Ripoll et al., 2015; Albert et al., 2016). The model was able to reproduce fairly well the genetic variabilities observed in the population and the WD effects, after the calibration of eight genotypic parameters, which are related to cell expansion, water transport, and sugar uptake. These three processes were strongly discriminant in the RIL population (Figure 6), and appeared as main traits to be considered in the future for breeding tomato adapted to WD conditions. Interestingly in the population, a few genotypes (among which Cervil) reached comparable fresh and dry masses under C and WD conditions. All these genotypes are in the range 5–20 g FW and 9–12% dm (Figure 2), but they clustered in groups 2, 4, and 5, whereas Cervil was in group 1 (Figure 6). Thus, the low sensitivity to WD could not be related to one single parameter/process, and a more detailed phenotyping of these RILs at the plant and fruit levels could be useful to identify the main discriminating traits. According to the PCA on the calibrated genotypic parameters, conductivities merit special attention since high conductivities were associated with high dry matter content and heavy fruit weights. The sugar active uptake seemed to play an important role as well: the higher the maximum uptake rate was (nuM), the lower the decrease in fresh mass, which was probably associated with osmotic regulations.

The mean NRMSE value of the population was around 17%, which is quite performing (Table 2). The worst value was obtained for Levovil, but the fitting were largely improved when predictions were done independently under C or WD conditions (not shown), suggesting that some WD effects were poorly taken into account in this case. During the calibration step, the WD treatment was taken into account through several plant and fruit variables, i.e., the stem water potential, the initial dry and fresh masses, the sugar concentration of the dry matter, the fruit conductance involved in transpiration and the fruit osmotic pressure related to soluble components other than sugars. These variables were measured experimentally. On the contrary, other parameters were fixed since they cannot be easily measured. In the future, these parameters could be more deeply investigated. For instance, the impact of drought on phloem transport has been nicely illustrated through current model hypotheses (Sevanto, 2014). Accordingly, in the Virtual fruit model, the assumptions of impermeable conduit walls in the fruit pedicel and semipermeable walls in the fruit cells, implicitly involve that phloem transport in the pedicel is vulnerable to the increase of viscosity and to the geometry (number and size) of the conducting vessels. Sevanto (2014) demonstrated that wider or more numerous conduits are required to compensate for the increase in sap viscosity in order to maintain phloem transport under drought. In the present study, WD effects on these two parameters were overlooked. Indeed, in the absence of experimental value, the sugar concentration in phloem sap (Cp) was supposed to be constant over fruit development and the surface (cm2) of exchange of the vascular networks entering the fruit, was assumed to be proportional to the fruit surface area (Af), according to a non-dimensional constant coefficient, leading to smaller exchange area in case of the WD treatment. Thus, the conductivity of the phloem and xylem in the pedicel (lp1 and lx1) or in the fruit (lp and lx), which were estimated, likely integrated several properties of the conducting tissues. Both in the calibrating step (Figure 6) and the ideotype design (Figure 8), these parameters were highly discriminant and undoubtedly involved in the reduction of fresh weight loss under WD. So sugar concentration in the phloem and geometry of conducts appeared as important components of the water deficit adaptation strategies, which have to be more deeply investigated as well as their genetic variability.

The maximum cell wall extensibility (phiMax) also appeared as a discriminant parameter in the population. Despite the growing number of studies and methods to investigate cell wall extensibility and elasticity (Cosgrove, 2016), data are currently missing to parameterize fruit models. In the present model, cell wall extensibility decreases exponentially with time from phiMax, which was considered to be genotype dependent, whereas the rate of decrease was constant and taken from Liu et al. (2007). In the RIL population, phiMax was negatively correlated with the maximum rate of sugar active uptake (nuM) and was associated with high loss of fresh mass under WD, likely because the large fruit-size genotypes (mainly Lev) combined both traits in this population.

### QTL analysis of model genotypic parameters

The added value of QTLs for model parameters lies in their expected stability, on the contrary to other QTLs for phenotypic traits, which fluctuate with environmental conditions. We detected QTLs for six of the 10 genotypic parameters and two PCA axes, four of them (for Lp1, Lx1, and Lp) with a significance level below 0.1. The QTLs were located in four chromosomic regions. The QTL for lp1 on chromosome 2 colocalized with QTLs detected in Albert et al. (2016) for plant fruit number (constitutive effect under control and WD conditions), fruit firmness (constitutive effect under control and WD conditions), dm (specific to control condition) and soluble solid content (SSC, with changing effect according to the watering regime). Besides, this QTL was present in the genomic region carrying the cloned tomato fresh weight QTL FW2.2 (Frary et al., 2000). Close to this QTL, several QTLs for sugar content were fine mapped (Lecomte et al., 2004) The QTLs for rxp, lx1 and axis3 in the centromeric region of chromosome 4 colocalized with a QTL for fruit firmness (FIR, specific to the WD condition) detected in Albert et al. (2016); this connection between conductivity and firmness under WD may result from the effect of turgor regulation on fruit firmness (Shackel et al., 1991). QTLs detected for nuM and axis 2 (related to sugar active uptake) did not colocalize with any QTL identified in Albert et al. (2016). However, QTL for soluble solid content were identified in this genomic region in other tomato populations (Pascual et al., 2016). Finally, the QTLs for lp and lx on top of chromosome 8 colocalized with QTLs for flowering time (Flw, specific to WD condition), fruit pH (specific to WD condition), vitamin C content in fruit on a fresh weight and on a dry weight basis (WD specific and constitutive, respectively) and yield (with changing effect according to the watering regime). Unfortunately the confidence intervals were too large to check for candidate genes, but future studies should more deeply investigate these regions, in particular regarding the pedicel and fruit conductivities.

Interestingly, the seven genotypes belonging to group 1 in the PCA (Figure 6) all carried the Cervil allele for the lp and lx QTLs on chromosome 8. Besides, these genotypes also carried the Cervil allele for a yield QTL detected by (Albert et al., 2016) on chromosome 4, close to the QTLs identified for rxp and lx1 (3.16 Mbp upper on the chromosome). No specific allele at the QTL was identified for the other groups of the PCA. We detected only one QTL for one of the four genotypic parameters related to sugar uptake, hypothetically due to their skewed distribution and to the estimation error. In the Fruit model, sugars may be allocated to fruit through passive diffusion, mass flow or active transport, the last being the most discriminant in our population. Different transporters are required for efficient phloem unloading in fruit pericarp at the rapid expansion phase (Ruan and Patrick, 1995), operating with different energetic and kinetic constraints (reviewed by Osorio et al., 2014). In the model, all these transporters are compiled into one single Michaelis-Menten function, which might explain that no specific QTL was detected.

### Ideotypes of tomato adapted to WD depending on trade-offs between fresh weight and dry matter content

In the light of experimental data, one challenge for producing tomatoes under WD conditions will be to avoid the reduction of fresh mass of large fruited genotypes, while maintaining or increasing the fruit dm content, which correlates with the accumulation of sugars and acids, both involved in fruit taste. In confront to previous works (Semenov et al., 2014; Rötter et al., 2015), the problem was complex, first, because the process-based model used in this study was relatively sophisticated, second, because we aimed at maintaining quality and increasing yield under WD conditions. We were able to design large-fruited ideotypes rich in dm (9% dm content) and with reasonable fresh mass loss under WD (<20%) which outperformed Levovil (60% fresh mass loss and 6% dm content). Pedicel conductivity and fruit composite membrane conductivity were opposed in the ideotype population (Figure 8). The model considers three pathways for water and carbon flows: the plant-to-pedicel, the pedicel-to-fruit and the fruit apoplasm-to-cell, which all differ in carbon concentration and water potential. Conductance is mainly axial in the first two pathways (plant-pedicel and pedicel-fruit), whereas it is radial within the fruit. Thus high conductance in the pedicel which promotes water and sugar inflows in combination with high active uptake of sugars could be a successful strategy to produce large fruit-size ideotypes able to maintain, under WD conditions, a fresh weight above 100 g and dm content above 6 % (group 1 on Figure 8). These ideotypes also exhibit a low tauA value, indicating that the active uptake decreases slowly during the growth stage. On the contrary, the medium fruit-size ideotypes (group 2) were associated with low pedicel conductance and sugar uptake rate, but high fruit composite membrane conductivity. Such interactions between pedicel and fruit conductivities in relation to the demand for water and carbon, is intriguing and should deserve further attention. As mentioned above, the genetic variability of the conducting tissue geometry in the pedicel and fruit has been hardly described. In an anatomical descriptive study, Rančić et al. (2010) suggested that the low phloem efficiency (defined as the ratio between fruit dry weight and phloem pedicel area) of tomato flacca mutants was responsible for low fruit growth, whereas the phloem area and the functional xylem area were not affected compared to the wild type. In agreement, a modeling approach showed that, under a wide range of conditions, water import in young tomato fruit would be limited by the pedicel resistance (Bussières, 2002) and by phloem conductivity in relation to sap viscosity (Bussiéres et al., 2011). In this respect, QTL observed on chromosome 2, 4, and 8 may be really interesting. Regarding the small fruit-size genotypes, the four RILs, which scattered among the ideotypes (Figure 7C) could probably bring new interesting source of genetic variations for breeding programs, as their fruit fresh mass at maturity is two to three times higher than the fresh mass of Cervil, which is already known to be WD resistant (Albert et al., 2016; Ripoll et al., 2016).

Comparing the RIL population (Figure 6) with the ideotype population (Figure 8), a main difference states in the orthogonality of nuM and lx/lp or lx1/lp1 in the RIL population, suggesting that fruit growth was limited either by the incoming fluxes (group 5 on Figure 6) or by the active transport of sugars (Lev or group 1 on Figure 6). Thus, the uptake of carbon was likely the limiting step for fruit growth of large fruit-size genotypes such as Lev, which bears large fruits with low dry matter content in the C condition.

## Conclusions

The fruit model was able to reproduce contrasting behaviors in the RIL population, regarding fresh weight loss and/or dm content increase under WD. Cell expansion, water transport and sugar uptake were all involved in the genetic variability of the fruit response to WD, but pedicel conductivity and active uptake of sugars seemed to be the key-mechanisms. In the future, model improvements should account for WD effects on cell wall extensibility, sugar uptake and exchanges of water between phloem and xylem tissues. Such advances will boost our understanding of the complex interactions between osmotic adjustments, changes in cell wall extensibility and maintenance of cell turgor under WD. The present study also outlined three interesting QTLs that deserve attention in breeding program for adaptation to WD and 4 RILs, which could bring new interesting traits in this regard. Finally, we are aware of the fact that Levovil is the only big fruit size genotype in the studied population; all other RILs ranged between 5 and 60 g FW. Thus, applying our approach to other tomato populations will be valuable. In a longer-term perspective, using a plant-fruit model (Baldazzi et al., 2013) would allow a better assessment of the respective contribution of source and sink capacities to the genetic variability. From a methodological point of view, other algorithms e.g., the Reference-point-based Non-dominated Sorting Genetic Algorithm R-NSGA-II could be used to calibrate the process-based model considering individual errors for each output variables, which could open new perspectives regarding the accurate integration of genetic information into the process-based model. Indeed, here ideotypes were discussed without considering the genetic constraints (epistasis or linkage). For this purpose, we should first integrate the genetic information into the process-based model through the QTL analysis. Then, we shall consider the allelic combinations of the loci involved in the genetic models allowing the computation of the parameter values. In this step, taking into account the genetic constraints (probabilities of two loci to be identical) shall be achieved either through their direct integration into the genetic model or through the optimization algorithm (mathematical formulation of the problem). In the future, such genetic models could be used to test virtual scenarios of fruit adaptation to water stress and identify key-regions on the tomato genome.

## Author contributions

NB, MG, GV, and MM conceived and designed the work; BB and NB collected the experimental data; DC, PV, and MM devised the algorithms and performed the simulations; DC, NB, MG, GV, EA, MC, and MM analyzed and interpreted the data; DC, NB, MM, GV, and EA wrote the paper; MG, MC, and VB revised it critically; all authors approved the final version.

## Funding

INRA provided all experimental and modeling supports as well as permanent manpower.

### Conflict of interest statement

The authors declare that the research was conducted in the absence of any commercial or financial relationships that could be construed as a potential conflict of interest.
